# Abnormal Response of the Proliferation and Differentiation of Growth Plate Chondrocytes to Melatonin in Adolescent Idiopathic Scoliosis

**DOI:** 10.3390/ijms150917100

**Published:** 2014-09-25

**Authors:** William Wei-Jun Wang, Gene Chi-Wai Man, Jack Ho Wong, Tzi-Bun Ng, Kwong-Man Lee, Bobby Kin-Wah Ng, Hiu-Yan Yeung, Yong Qiu, Jack Chun-Yiu Cheng

**Affiliations:** 1 Department of Spine Surgery, Drum Tower Hospital, Nanjing University Medical School, Nanjing 210008, China; E-Mails: drwilliamwang@163.com (W.W.-J.W.); scoliosis2002@sina.com (Y.Q.); 2 Department of Orthopaedics and Traumatology, the Chinese University of Hong Kong, Hong Kong, China; E-Mails: bobng@ort.cuhk.edu.hk (B.K.-W.N.); scoliosis2002@sina.com (H.-Y.Y.); 3 The Joint Scoliosis Research Center of the Chinese University of Hong Kong–Nanjing University, Hong Kong, China; 4 Department of Obstetrics and Gynaecology, Faculty of Medicine, the Chinese University of Hong Kong, Hong Kong, China; E-Mail: geneman@cuhk.edu.hk; 5 School of Biomedical Sciences, the Chinese University of Hong Kong, Hong Kong, China; E-Mails: jack1993@yahoo.com (J.H.W.); tzibunng@cuhk.edu.hk (T.-B.N.); 6 Lee Hysan Clinical Research Laboratory, the Chinese University of Hong Kong, Hong Kong, China; E-Mail: simonlee@cuhk.edu.hk

**Keywords:** idiopathic scoliosis, melatonin, growth plate, chondrocytes, proliferation, differentiation, MT2 receptor, antagonist, dysfunction

## Abstract

Abnormalities in the melatonin signaling pathway and the involvement of melatonin receptor MT2 have been reported in patients with adolescent idiopathic scoliosis (AIS). Whether these abnormalities were involved in the systemic abnormal skeletal growth in AIS during the peripubertal period remain unknown. In this cross-sectional case-control study, growth plate chondrocytes (GPCs) were cultured from twenty AIS and ten normal control subjects. Although the MT2 receptor was identified in GPCs from both AIS and controls, its mRNA expression was significantly lower in AIS patients than the controls. GPCs were cultured in the presence of either the vehicle or various concentrations of melatonin, with or without the selective MT2 melatonin receptor antagonist 4-P-PDOT (10 µM). Then the cell viability and the mRNA expression of *collagen type X* (*COLX*) and alkaline phosphatase (*ALP*) were assessed by MTT and qPCR, respectively. In the control GPCs, melatonin at the concentrations of 1, 100 nM and 10 µM significantly reduced the population of viable cells, and the mRNA level of *COLX* and *ALP* compared to the vehicle. Similar changes were not observed in the presence of 4-P-PDOT. Further, neither proliferation nor differentiation of GPCs from AIS patients was affected by the melatonin treatment. These findings support the presence of a functional abnormality of the melatonin signaling pathway in AIS GPCs, which might be associated with the abnormal endochondral ossification in AIS patients.

## 1. Introduction

Adolescent idiopathic scoliosis (AIS) is a three-dimensional structural deformity of the spine that occurs during the peripubertal period [1]. Although no consensus has been reached on its etiology, one of the generally accepted concepts is the presence of abnormal skeletal growth in AIS [2,3]. This abnormality is manifested during the peripubertal period in patients with AIS in that they tend to be taller, leaner and have a longer arm span than their healthy peers [4,5]. A faster growth of AIS subjects during puberty was also recorded in longitudinal studies [6]. Studies on magnetic resonance images disclosed a related anterior spinal overgrowth in girls with AIS [7,8]. Furthermore, a histomorphometric study of the vertebral endplate revealed more active growth in the anterior than the posterior spinal column in AIS patients [9]. All of these observations indicated the presence of an abnormal systemic growth and the likelihood of an abnormal regulation and modulation of skeletal growth and endochondral ossification in patients with AIS.

Growing interest has arisen in the past decades following the report of “idiopathic-like” scoliosis in animals with pinealectomy-induced melatonin deficiency [10,11,12]. Although considerable controversies still exist on whether an abnormal plasma melatonin level is present in patients with AIS [13,14,15], only a few studies focused on examining abnormalities in the signaling pathway of melatonin rather than the circulating melatonin level [16,17,18]. Melatonin failed to inhibit the increase of 3',5'-cyclic adenosine monophosphate (cAMP) induced by forskolin in osteoblasts from AIS patients when compared with cells from normal control subjects [16,18]. In addition, abnormality in the genotypic frequency at the promoter region of the *MT2* gene, which was detected in a genetic association study [19], indicated that the *MT2* gene was likely a predisposition gene for AIS, although there were controversies [20,21,22,23]. Furthermore, the osteoblasts from girls with AIS exhibited an abnormal response to melatonin in terms of proliferation and differentiation [24], which might be due to the abnormalities in MT2 receptor expression [25].

The functional outcome of abnormalities in the melatonin signaling pathway in the regulation of endochondral ossification in AIS, however, has not been reported. It has been documented that melatonin inhibited both proliferation and differentiation of rat vertebral body growth plate (VBGP) chondrocytes [26] with the involvement of MT1 and MT2 receptors. In addition, a recent study showed that melatonin enhanced the chondrogenic differentiation of human mesenchymal stem cells [27], mediated at least partially by the two membrane melatonin receptors. We hypothesized that melatonin could be involved in the regulation and modulation of endochondral ossification in human, and that abnormality in the melatonin signaling pathway could contribute to the abnormal skeletal growth in AIS subjects. The proposed study aimed to investigate the expression of the melatonin membrane MT2 receptor in human growth plate chondrocytes (GPCs) and also the effect of melatonin on the proliferation and differentiation of GPCs isolated from AIS patients and normal control subjects, and cultured *in vitro*.

## 2. Results and Discussion

### 2.1. Expression of MT2 Receptor in GPCs

**Figure 1:**
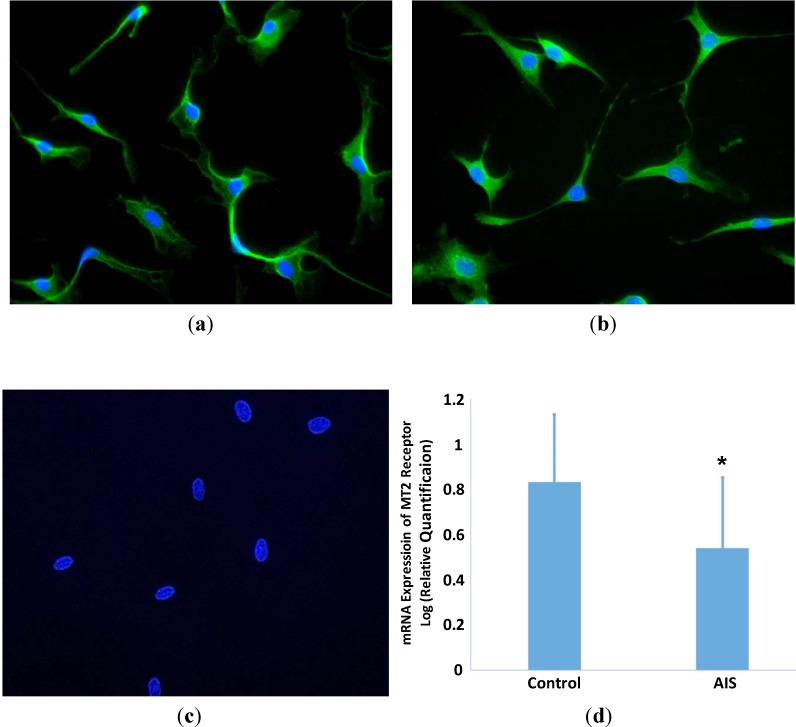
MT2 receptor expression in growth plate chondrocytes (GPCs) from adolescent idiopathic scoliosis (AIS) patients and control subjects. Immunofluorescent staining was carried out using purified rabbit polyclonal anti-MT2 antibodies. MT2 receptor was demonstrated in GPCs from AIS patients (**a**) and control (**b**) subjects; The immunoreactivity was observed mainly in the cytoplasm. No staining was observed in the negative control (**c**); The mRNA expression of *MT2* receptor was quantified by qRT-PCR. The GPCs from AIS patients showed a significantly lower expression than those of control subjects (**d**) (Students’ *t* test, * *p* < 0.05).

In both AIS and control groups, MT2 receptors were expressed mainly in the cytoplasm and not in the nuclei of GPCs (Figure 1). No positive signal was observed in the absence of the primary antibody for both receptors. In addition, the mRNA expression of MT2 receptor in GPCs of AIS subjects, determined by quantitative real time-polymerase chain reaction (qRT-PCR) was significantly lower than that in control GPCs (*p* < 0.05)

### 2.2. Effect of Melatonin on the Proliferation and Differentiation of Growth Plate Chondrocytes (GPCs) from Normal Control Subjects

**Figure 2:**
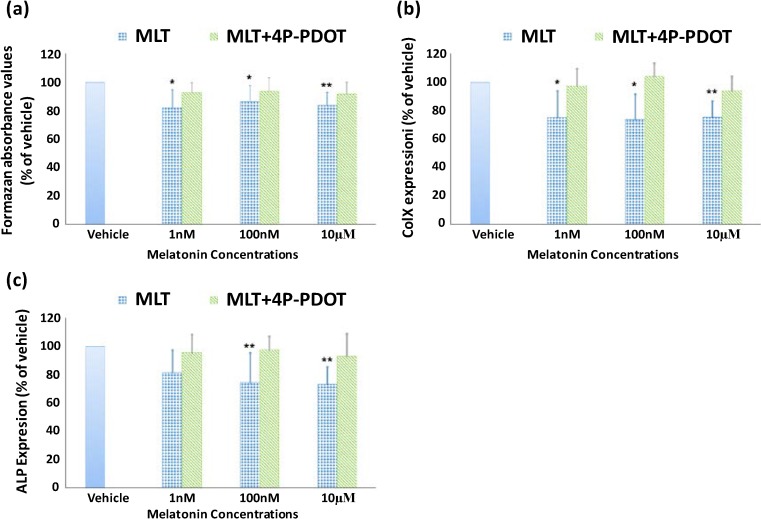
Effect of melatonin on the proliferation and gene expression of collagen type X (*COLX*) and alkaline phosphatase (*ALP*) in cultured GPCs from control subjects. Data represent mean ± standard deviation (*n* = 10) The cell viability at the melatonin (MLT) concentration of 1, 100 nM and 10 µM was 82.2% ± 12.3%, 86.4% ± 11.0% and 83.8% ± 9.1% of that in the vehicle group, respectively. Significant differences were retrieved by One-sample *t*-test (* *p* < 0.05, ** *p* < 0.01) (**a**); The expression of *COLX* (**b**) and *ALP* (**c**) was also reduced by melatonin especially at high concentrations. However, these inhibitory effects were reversed in the presence of 4-P-PDOT (10 µM).

To minimize the variation of cell activity among different subjects, the results were expressed as the percentage of the vehicle control. After treatment for three days with melatonin at the concentrations of 1, 100 nM and 10 µM, the percentage of viable growth plate chondrocytes (GPCs) from control subjects was reduced significantly by 17.8% ± 12.3% (*p* < 0.05), 13.6% ± 11.0% (*p* < 0.05) and 16.2% ± 9.1% (*p* < 0.01) (mean ± standard deviation, *n* = 10), respectively, compared to the vehicle group (Figure 2). In the presence of 4-phenyl-2-propionamidotetralin (4-P-PDOT, 10 µM), however, the percentage of viable cells was 92.6% ± 7.0%, 93.5% ± 9.6% and 91.7% ± 8.3% of the vehicle group at 1, 100 nM and 10 µM concentrations of melatonin, respectively (*p* > 0.05). In addition, the mRNA level of *collagen type X* (*COLX*) was reduced by 24.7% ± 18.9% (*p* < 0.05), 25.9% ± 17.5% (*p* < 0.05) and 24.5% ± 11.4% (*p* < 0.01), and alkaline phosphatase (ALP) was reduced by 18.7% ± 16.3%, 25.7% ± 21.2% (*p* < 0.01) and 26.8% ± 12.5% (*p* < 0.01) in the presence of 1, 100 nM and 10 µM melatonin, respectively. Furthermore, the effect of melatonin was reversed in the presence of 4-P-PDOT.

To the best of our knowledge, the current study constitutes the first report on the inhibitory effect of melatonin on the proliferation and differentiation of GPCs in human. In an *in vitro* study, melatonin at high concentrations (10, 100 µg/mL) showed an inhibitory effect on both the proliferation and differentiation of cultured rat vertebral body growth plate chondrocytes [26]. After incubation for 24 h in medium containing melatonin, the cell proliferation, gene expression of collagen type II and aggrecan, as well as protein expression of proliferating cell nuclear antigen (PCNA), Sox9 and Smad4 were significantly reduced. Moreover, it was found that the effects of melatonin could be reversed by the melatonin receptor antagonist luzindole, indicating the involvement of membrane melatonin receptors in these functions [26]. In a broiler chicken model, Aota *et al.* [28] noted that pinealectomized chickens had a significantly increased area of labeled hypertrophic zone per total hypertrophic zone compared with control chickens (32.8% ± 12.5% *vs.* 6.4% ± 5.0%, *p* < 0.005), as well as an increased number of hypertrophic and proliferative chondrocytes. In the present study, the GPCs were treated with melatonin in either physiological (10^−9^ M) or pharmacological concentrations (10^−7^, 10^−5^ M). The results showed that melatonin inhibited both the proliferation and differentiation of GPCs, in a dosage-dependent manner and the effects were even more significant with an increase of the melatonin concentration (cell viability was not significantly inhibited by melatonin at a concentration of 0.01 nM; data not shown). It is interesting to note that in a study conducted by Zhong *et al.* [26] that melatonin significantly inhibited the proliferation and differentiation of VBGP chondrocytes at high dosages (10 and 100 µg/mL, or 43 and 430 µM) but not at low dosages (0.1 and 1 µg/mL, or 0.43 and 4.3 µM), while in the present study, melatonin at 1 nM and 0.1 µM concentrations demonstrated an inhibitory effect on both proliferation and differentiation of human GPCs. These differences might be due to the different study protocols employed. In the study performed by Zhong *et al.* [26] the chondrocytes were treated with melatonin for 24 h only, while in our study the GPCs were cultured with melatonin for 72 h in view of the doubling time of GPCs being around 48 h in our study (data not shown).

One of the signaling pathways regulating chondrocyte proliferation and differentiation is mediated through G proteins. The activation of Gs alpha-subunit would stimulate the cAMP-protein kinase A pathway [29] subsequently resulting in phosphorylation of SOX9 and expression of Col2α1 which would lead the chondrocytes to continue to proliferate without going into the normal differentiation phase [30,31]. On the other hand, it has been shown that melatonin, acting through the melatonin receptors, high-affinity GPCR MT1 and MT2, activates the Gi alpha-subunit and, in turn, inhibits cAMP accumulation [32]. It is likely that in the current study, melatonin might exert its action by activating membrane receptors to inhibit cAMP accumulation. This was supported by the identification of the expression of the MT2 receptor in human GPCs, and the finding that the inhibitory effects of melatonin on both proliferation and differentiation of GPCs in control subjects could be reversed by 4-P-PDOT. Similar findings on rat VBGP have been reported [26]. Moreover, the hypothesized signaling pathway of melatonin was further supported by the finding of reduced protein expression of Sox9 in VBGP following melatonin treatment [26].

### 2.3. Lack of Response of GPCs to Melatonin Treatment in Both Proliferation and Differentiation in AIS Patients

**Figure 3:**
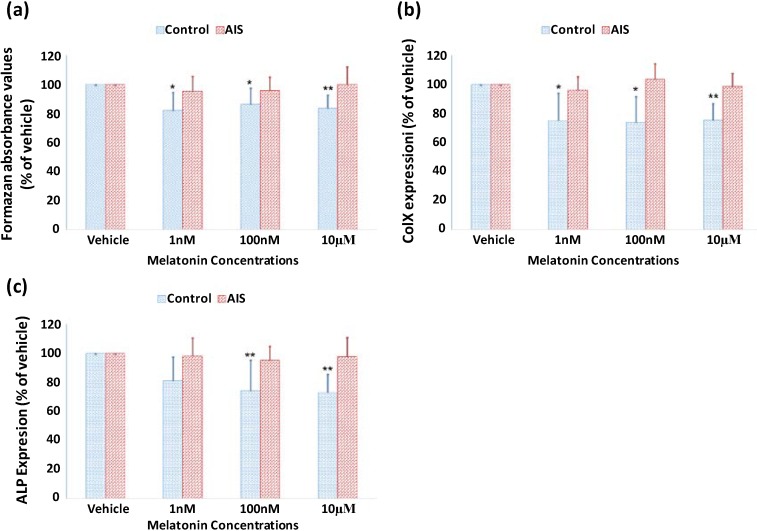
Lack of effect of melatonin on the proliferation and differentiation of GPCs from AIS patients. GPCs from 20 AIS patients and 10 control adolescents were isolated and cultured. Data represent mean ± standard deviation. The GPCs from AIS patients showed no response to the melatonin treatment in proliferation (**a**); and gene expression of *COLX* (**b**) and ALP (**c**). (* *p* < 0.05, ** *p* < 0.01).

In patients with AIS, the effect of melatonin on proliferation in GPCs was observed as the percentage of viable GPCs was 95.3% ± 10.3%, 96.0% ± 9.1% and 100.0% ± 12.3% of that in the vehicle group at the melatonin concentration of 1, 100 nM and 10 µM, respectively. No significant difference was found between the vehicle and melatonin treatments (Figure 3). Furthermore, the expression of *ALP* and *COLX* was not affected in the presence of the different dosages of melatonin.

In AIS, the lack of response of GPCs in both proliferation and differentiation to melatonin might be attributed to derangements in the signaling pathway of melatonin. A systemic melatonin signaling pathway dysfunction has been described by Moreau *et al.* [17] The dysfunction was shown to be related to abnormalities in the phosphorylation of Gi protein [16]. It suggested a switching of MT2 receptor coupling from Gi alpha-subunit to Gs alpha-subunit [18]. *MT2* gene polymorphism has been reported to be statistically associated with the occurrence of AIS suggesting that MT2 might be a susceptibility gene [19]. In the present investigation, the expression of MT2 receptor in GPCs from AIS and control subjects was demonstrated by immunofluorescence but there were no significant qualitative differences between AIS and control subjects. However, another study indicated that it was likely that the mRNA expression of MT2 receptor was significantly reduced in GPCs from AIS when compared with control subjects [33]. In osteoblasts from girls with AIS, an abnormal expression of MT2 receptor has been observed, and the expression was not demonstrable in four out of the eleven girls [25]. Furthermore, AIS patients with a low level of expression of MT2 receptor in osteoblasts showed a longer arm span than those with a normal expression level of MT2 receptor [34]. Hence, a comprehensive investigation on the MT2 receptor including the expression of RNA and protein, the structure of DNA and protein, and the coupling between MT2 receptor and Gi/Gs proteins should be carried out in GPCs from AIS subjects and control subjects to facilitate further understanding of the abnormalities in the MT2 receptor.

In a histomorphometric study on the longitudinal bone growth in pinealectomized chickens, an increased number of hypertrophic and proliferative chondrocytes was observed compared to the control [28]. Although longitudinal growth was not measured due to the multiple labeling techniques used, the authors believed that the enlarged endochondral bone coverage could further minimize metaphyseal bone production, leading to a rapid and marked loss of cancellous bone volume, and this would accelerate bone elongation economically [28]. The lack of response of GPCs to the action of melatonin in regulating proliferation and differentiation could be linked to the abnormal endochondral ossification, which affects skeletal growth in AIS. Abnormal endochondral ossification has been speculated in patients with AIS [4,7,8] and thought to be a contributing factor in the etiopathogenesis of the disease. The impaired ability of GPCs in AIS patients to respond to the inhibitory effect of melatonin on chondrocyte proliferation and differentiation found in the current study indicates a role of melatonin in the regulation and modulation of endochondral ossification. Without the normal response to the inhibitory action of melatonin, the GPCs were speculated to proliferate. This, in turn, affects the growth activity as suggested in the previous study [9].

In addition to its effect on chondrogenesis, melatonin has shown a significant effect on osteogenesis. Melatonin has been found to induce osteogenesis of human mesenchymal stem cells [35], and the enhanced alkaline phosphatase activity in osteogenic medium could be reduced by the presence of 4-P-PDOT, the selective MT2 receptor antagonism [36]. For osteoblasts, melatonin could enhance the proliferation of osteoblasts *in vitro* at pharmacological doses [37] but not at physiological doses [38,39]. In addition, Satomura *et al.* [37] demonstrated the dose-dependent stimulation of alkaline phosphatase activity in human osteoblasts by melatonin. Furthermore, melatonin enhanced mineralized matrix formation *in vivo* [37,40]. In patients with AIS, the abnormal response of osteoblasts to melatonin has also been reported recently. Man *et al.* [24] noted that melatonin failed to promote both proliferation and differentiation of osteoblasts from AIS subjects, which might contribute to the low bone mineral density in these patients [41,42,43,44]. This abnormality of melatonin could be attributed to the abnormal expression of MT2 receptor [25,34]. Taking these observations together, it is likely that melatonin signaling pathway dysfunction could be a systemic problem and plays an important role in the abnormal systemic bone growth in AIS subjects [4,42,43]. This was also supported by the finding of the disrupted cartilage matrix and the rapid and marked loss of cancellous bone volume in chickens which had undergone pinealectomy [28].

A limitation of the present study is that the sites at which the cartilage specimens were collected varied because of the small number of subjects who could be recruited and the ethical problem and difficulty encountered in harvesting growth plate cartilages. By isolating GPCs from growth plates and culturing the cells in a standard environment, however, the environmental factors affecting the biology of chondrocytes could be minimized. Although functional abnormalities of the melatonin signaling pathway in regulating the GPCs from AIS patients were detected, the underlying pathogenesis involving the MT2 receptor and Gi protein has not been fully elucidated. As the exact mechanism of the dysfunction in melatonin signaling cannot be fully uncovered in this study, further investigations on the role and mechanism of MT2 receptor in AIS patients are warranted. Furthermore, although comparisons on the proliferation, mRNA expression of MT2 receptor, *COLX* and *ALP* of GPCs between AIS and control subjects would also advance our understanding of the endochondral ossification activity in AIS patients, this was not carried out in the present study. We had the concern that *in vitro* culture of GPCs might lead to changes in the cell viability and mRNA expression of *COLX* and *ALP* from the original status in cartilage. Hence, it would be best to extract RNA and protein from the cartilage samples to carry out such assays.

## 3. Experimental Section

### 3.1. Reagents

Dulbecco’s modified Eagle’s medium (DMEM), fetal bovine serum (FBS), penicillin, streptomycin and neomycin (PSN), ascorbic acid, and Alexa Fluor-488 goat anti-rabbit IgG were obtained from Invitrogen (Grand Island, NY, USA). Melatonin, trypsin, hyaluronidase, collagenase II, dimethyl sulfoxide (DMSO), 3-(4,5-dimethylthiazol-2-yl)-2,5-diphenyltetrazolium bromide (MTT) and bovine serum albumin (BSA) were obtained from Sigma–Aldrich (St. Louis, MO, USA). Culture flasks and culture plates were supplied by Corning Life Science (Oneonta, NY, USA). Rabbit anti-MT2 affinity-purified polyclonal antibodies were obtained from Chemicon (Temecula, CA, USA). VECTASHIELD mounting medium with DAPI were obtained from Vector Laboratories (Burlingame, CA, USA). RNeasy Mini Kit was purchased from Qiagen (Hilden, Germany), Moloney murine leukemia virus reverse transcriptase was bought from Promega (Tokyo, Japan) and SYBR Green master mix was obtained from Roche Applied Science (Indianapolis, IN, USA)

### 3.2. Recruitment of Subjects

Twenty AIS patients (seventeen girls and three boys) aged 13.8 ± 3.4 years, with a severe curvature of the spine as evidenced by Cobb angle ranging from 45° to 95°, and undergoing corrective spinal surgery with either anterior spinal fusion (*n* = 5) or posterior spinal fusion (*n* = 15), were recruited. Ten non-AIS subjects (six girls and four boys) with a mean age of 13.7 ± 2.8 years, and either developmental dysplasia of the hip, lumbar disc herniation, or trauma, and undergoing other forms of orthopaedic surgery, were recruited as control subjects. Subjects with other forms of spinal deformity, abnormal metabolic or melatonin-related diseases, such as sleeping problems, skin pigment anomalies, and endocrine disorders, were excluded [45]. Standardized growth plate cartilage biopsies were harvested intra-operatively from the vertebral end plate, spinal process or iliac crest apophyses in the AIS group and from the iliac crest apophyses in the control group. The study was approved by the University Clinical Research Ethics Committee (Ref. CRE-2007.123; 3 April 2007; Joint The Chinese University of Hong Kong—New Territories East Cluster Clinical Research Ethics Committee, Hong Kong, China) and written informed consent had been obtained from the subjects before commencement of the study.

### 3.3. Primary Culture of GPCs

The methods of Lee *et al.* and Hidvegi *et al.* [46,47] were followed with minor modifications. Growth plate biopsies were cleaned from the attached connective tissue under sterile conditions. After cutting into small pieces (around 1 × 1 mm), the cartilage samples were subjected to a series of enzyme digestions to isolate GPCs from the cartilage matrix [46,47]. The pieces of cartilage were incubated in the presence of trypsin (1 mg/mL, 10 mL/g cartilage) for 10 min, hyaluronidase (1 mg/mL, 10 mL/g cartilage) for 30 min, and collagenase II (0.5 mg/mL, 20 mL/g cartilage) for 4–6 h. GPCs were then cultured, at 37 °C in a humidified atmosphere of 5% CO_2_, in a monolayer in DMEM supplemented with 10% FBS and 100 U/mL PSN. The medium was refreshed every 2–3 days.

The morphology of GPCs in culture was observed under an inverted microscope and captured with the equipped camera. Chondrocytes from both AIS patients and control subjects displayed a fibroblast-like and flattened appearance when adhered to the bottom of the culture flask. After culture for another two weeks, the cells assumed a more polygonal shape. There was no discernible difference between GPCs from AIS and control subjects. GPCs at the end of the second passage were harvested and used for further assays.

### 3.4. Expression of Melatonin Membrane MT2 Receptor

The expression of MT2 receptor on GPCs was determined by immunofluorescent staining. GPCs were seeded on cover slips coated with poly-l-lysine and then cultured overnight for attachment. After washing in phosphate-buffered saline (PBS), the samples were fixed in acetone for 30 min. After eliminating non-specific binding by exposure to 5% goat serum in 1% BSA/PBS at room temperature for 20 min, the samples were incubated overnight with the primary antibody at 4 °C in a moist chamber. Rabbit anti-MT2 affinity-purified polyclonal antibodies (1 mg/mL) were diluted in blocking buffer (1% BSA/PBS) (1:100, *v*:*v*). Then samples were incubated in the presence of Alexa Fluor-488 goat anti-rabbit IgG diluted with blocking buffer (1:100, *v*:*v*) at room temperature for 1 h. The cover slips were stained with DAPI in VECTASHIELD mounting medium and mounted on glass slides with nail polish. Immunofluorescence was observed with a fluorescence microscope (LEICA DM RXA2, Leica Microsystems, Wetzlar, Germany).

To quantify the expression of MT2 receptor, GPCs were harvested and then RNA was extracted by using the RNeasy Mini Kit (Qiagen, Hilden, Germany) according to the manufacturer’s instructions. Sample mRNA was quantified by measuring the optical density at 260 and 280 nm. Extracted total RNA (1 µg) was reverse-transcribed with Moloney murine leukemia virus reverse transcriptase (Promega) as detailed in the manufacturer’s guidelines. qRT-PCR was performed in a total volume of 10 µL containing 5 µL diluted template DNA (1:50 dilution), 500 nM sense and antisense primers, 1 µL of SYBR Green master mix (Roche Applied Science), and 20 mM MgCl_2_. PCR amplification and quantification were performed in a Light Cycler Carousel-Based system (Roche Applied Science) as follows: denaturation for 10 min at 95 °C, followed by 45 amplification cycles (15 s at 95 °C for denaturation, 5 s for annealing at 56–58 °C, and 15 s for extension at 72 °C). The amount of RNA was calculated from the measured threshold cycles (*C*_t_) by employing a standard curve. The data were normalized by determination of the amount of glyceraldehyde-3-phosphate dehydrogenase (GAPDH). Sequences of the sense and antisense primers for MT2 receptor were 5'-CTCCCTATCGCTGTCGTGTC-3' and 5'-ATCTGGGGAGCCATTTCTTG-3' respectively [33]. The relative quantification values were expressed in log to obtain a normal distribution for analysis.

### 3.5. Effect of Melatonin on Proliferation of GPCs

Isolated GPCs were seeded in a 96-well plate at a density of 10,000 cells/ cm^2^ in culture medium. After incubation overnight, the cells were cultured in serum-free DMEM medium for 24 h. The GPCs were then cultured with DMEM containing 1% FBS, together with vehicle alone (DMSO, 10 µM) or in the presence of various concentrations of melatonin (1, 100 nM, and 10 µM). The final concentration of the vehicle was minimized to 10 µM. To further evaluate whether the effect of melatonin on the proliferation of GPCs was mediated by the MT2 receptor, the selective MT2 receptor antagonist 4-P-PDOT (10 µM, Tocris Cookson Inc., Ellisville, MO, USA) [48] was added together with the various concentrations of melatonin. The treatment was refreshed daily for three days. Then the cell viability was determined by the MTT assay in triplicate. Absorbance was measured by using a microplate reader at the wavelength of 570 nm with a reference wavelength of 630 nm.

### 3.6. Effect of Melatonin on Differentiation of GPCs

The GPCs were cultured until confluence, followed by switching to chondrocyte differentiation medium (MEM alpha medium supplied with 10% FBS and 1 mM glycerol-2-glycerophosphate, 100 U/mL PSN and 50 µg/mL ascorbic acid) [49]. Then the GPCs were treated with either vehicle or melatonin (1, 100nM and 10 µM), in the presence or absence of 4-P-PDOT for 14 days. The culture medium and melatonin were refreshed every other day. At the end of the treatment, the mRNA expression of *COLX* and *ALP* was determined by qRT-PCR.

GPCs were harvested and then RNA was extracted by using the RNeasy Mini Kit (Qiagen, Hilden, Germany) according to the manufacturer’s instructions. Sample mRNAs were quantified by measuring the optical density at 260 and 280 nm. Extracted total RNA (1 µg) was reverse-transcribed with Moloney murine leukemia virus reverse transcriptase (Promega) as detailed in the manufacturer’s guidelines.

qRT-PCR was performed in a total volume of 10 µL containing 5 µL diluted template DNA (1:50 dilution), 500 nM sense and antisense primers, 1 µL of SYBR Green master mix (Roche Applied Science), and 20 mM MgCl_2_. PCR amplification and quantification were performed in a Light Cycler Carousel-Based system (Roche Applied Science) as follows: denaturation for 10 min at 95 °C, followed by 45 amplification cycles (15 s at 95 °C for denaturation, 5 s for annealing at 56–58 °C, and 15 s for extension at 72 °C). The amount of RNA was calculated from the measured threshold cycles (*C*_t_) by employing a standard curve. The data were normalized by determination of the amount of glyceraldehyde-3-phosphate dehydrogenase (GAPDH). Sequences of the sense and antisense primers were 5'-CCAATGCCGAGTCAAA-3' and 5'-AGAGGCTTCACATACG-3' for *COLX*, 5'-CTCGTTGACACCTGGAAGAGCTTCAAACCG-3' and 5'-GGTCCGTCACGTTGTTCCTGTTCAGC-3' for ALP, and 5'-TGGTATCGTGGAAGGACTCATGAC-3' and 5'-ATGCCAGTGAGCTTCCCGTTCAGC-3' for GAPDH.

### 3.7. Statistical Analysis

All data were expressed as mean ± standard deviation (SD). SPSS/PC 13.0 (SPSS Inc., Chicago, IL, USA) was used for all statistical computations. Comparisons between vehicle and melatonin treatments were analyzed by one-sample *t*-test. Comparisons between AIS and control were made using independent samples *t*-test. A *p*-value smaller than 0.05 was considered statistically significant.

## 4. Conclusions

The present study represents the first report on the effect of melatonin on the proliferation and differentiation of GPCs from AIS and control subjects, signifying that melatonin is likely to be involved in the regulation and modulation of growth plate chondrocyte biology, which is important for endochondral ossification. The finding of the abnormal response of GPCs to melatonin in both proliferation and differentiation in AIS subjects provides further evidence supporting the presence of an abnormal systemic signaling pathway of melatonin in AIS patients, which might be linked to the abnormal endochondral ossification and skeletal growth.
